# PRECIOUS: PREvention of Complications to Improve OUtcome in elderly patients with acute Stroke—statistical analysis plan of a randomised, open, phase III, clinical trial with blinded outcome assessment

**DOI:** 10.1186/s13063-020-04717-0

**Published:** 2020-10-26

**Authors:** Jeroen C. de Jonge, Lisa J. Woodhouse, Hendrik Reinink, H. Bart van der Worp, Philip M. Bath, for the PRECIOUS investigators

**Affiliations:** 1 Department of Neurology and Neurosurgery, Brain Center, University Medical Center Utrecht, Utrecht University, Heidelberglaan 100, 3584 CX Utrecht, The Netherlands; 2 Stroke Trials Unit, Division of Clinical Neuroscience, University of Nottingham, Nottingham, UK; 3 Stroke, Nottingham University Hospitals NHS Trust, Nottingham, UK

**Keywords:** Stroke, Complications, Elderly, Ceftriaxone, Metoclopramide, Paracetamol

## Abstract

**Rationale:**

Aspiration, infections, and fever are common in the first days after stroke, especially in older patients. The occurrence of these complications has been associated with an increased risk of death or dependency.

**Aims and design:**

PREvention of Complications to Improve OUtcome in elderly patients with acute Stroke (PRECIOUS) is an international, multi-centre, 3 × 2 factorial, randomised, controlled, open-label clinical trial with blinded outcome assessment, which will assess whether prevention of aspiration, infections, or fever with metoclopramide, ceftriaxone, paracetamol, respectively, or any combination of these in the first 4 days after stroke onset improves functional outcome at 90 days in elderly patients with acute stroke.

**Discussion:**

This statistical analysis plan provides a technical description of the statistical methodology and unpopulated tables and figures. The paper is written prior to data lock and unblinding of treatment allocation.

**Trial registration:**

ISRCTN registry ISRCTN82217627. Registered on 22 September 2015. The trial was prospectively registered.

## Background

In the first days after stroke, about half of all patients develop one or more complications, including aspiration, infections, or fever. The risk of developing these events is greater in patients of higher age or with more severe stroke [1–3]. These complications can impede functional recovery, prolong hospital admissions, and are independently associated with an increased risk of death or long-term dependency [1, 2, 4–11]. The risk of developing these complications can be reduced by very simple, safe, and inexpensive measures, such as metoclopramide for the management of dysphagia, antibiotics for the prevention of infections, and paracetamol for the prevention of fever, but it is uncertain whether these measures also improve functional outcome [12–15]. In some generally small, randomised trials, preventive treatment with these drugs not only convincingly reduced the risks of aspiration, infections, or fever by one third to one half, but was also associated with clear trends towards a lower risk of death or poor outcome [12–15]. However, in two large randomised clinical trials, preventive treatment with antibiotics did not improve functional outcomes [16, 17]. Guidelines of the European Stroke Organisation concluded that there is insufficient evidence from randomised trials to make strong recommendations on whether, when, and to whom preventive antibiotic or antipyretic treatment should be given after ischaemic stroke or intracerebral haemorrhage [18, 19]. The PREvention of Complications to Improve OUtcome in elderly patients with acute Stroke (PRECIOUS) trial will assess whether prevention of aspiration, infections, or fever with metoclopramide, ceftriaxone, paracetamol, or any combination of these in the first 4 days after stroke onset improves functional outcome at 90 days in older patients with acute stroke. The current paper describes the statistical analysis plan (SAP) of the trial and conforms to the guidelines set by Gamble et al. [20]. The details of the study protocol of the PRECIOUS trial have been published earlier [21]. PRECIOUS has received funding from the *European Union’s Horizon 2020 research and innovation programme* under grant agreement no. 634809.

## Study methods

PRECIOUS is an international, multi-centre, multi-factorial, randomised, controlled, phase III, open-label clinical trial with blinded outcome assessment (PROBE). The primary objective is to assess whether prevention of aspiration, infections, or fever with metoclopramide, ceftriaxone, paracetamol, or any combination of these in the first 4 days after stroke onset improves functional outcome at 90 days in older patients with acute stroke. Patients will be randomly allocated in a 2 × 2 × 2 factorial design to any combination of open-label oral, rectal, or intravenous metoclopramide (10 mg thrice daily); intravenous ceftriaxone (2000 mg once daily); oral, rectal, or intravenous paracetamol (1000 mg four times daily); or usual care, started within 24 h after symptom onset and continued for 4 days or until complete recovery or discharge from hospital, if earlier. In patients with moderate to severe renal impairment or with severe hepatic impairment, the dose of metoclopramide is reduced to 5 mg thrice daily, and in patients with end-stage renal disease to 2.5 mg thrice daily. Patients will be stratified according to country (Estonia, Germany, Greece, Hungary, Italy, the Netherlands, Norway, Poland, UK), and there will be 5 minimisation factors: age (66–75 years; > 75 years), sex (male vs. female), stroke type (ischaemic stroke vs. intracerebral haemorrhage), stroke severity (NIHSS 6–12 vs. > 12), and diabetes mellitus (yes vs. no).A total of 3800 patients will be recruited, based on the sample size calculation described in the previously published protocol [21].

### Statistical interim analyses and stopping guidance

An independent Data and Safety Monitoring Board (DSMB) will conduct unblinded interim analyses after 600, 1200, 1800, 2400, and 3000 patients have completed follow-up to assess the safety of the interventions in the trial. With respect to efficacy, the DSMB will conduct unblinded interim analyses after 2400 patients had their final follow-up. DSMB members will receive listings of all SAE reports as well as unblinded aggregate summaries of data by treatment groups for review in closed meetings. The results of these interim analyses are confidential and limited to the members of DSMB.

### Timing of final analysis

This statistical analysis plan (SAP) will be signed off by the trial Steering Committee and then submitted for publication prior to data lock and final analysis. The final statistical analysis will be performed once recruitment has ceased, final follow-up and final outcome adjudication have been completed, final data have been checked and any errors corrected, and the database has been locked. The analyses will be carried out according to the current statistical analysis plan. The statistical analyses will be performed by the Nottingham Stroke Trial Unit (NSTU) at the University of Nottingham (UNOTT) in collaboration with the UMC Utrecht.

## Trial population

**Fig. 1:**
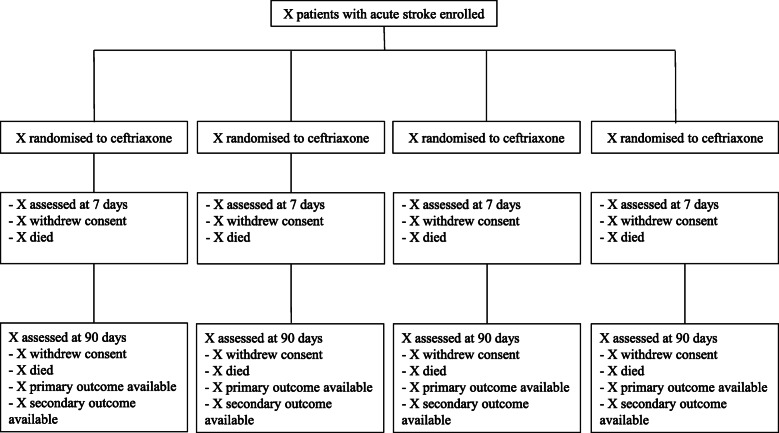
Trial profile

The study population will consist of patients aged 66 years or older who are hospitalised with moderately severe to severe (National Institutes of Health Stroke Scale (NIHSS) ≥ 6) acute ischaemic stroke or intracerebral haemorrhage. Patients will only be included if treatment can be started within 24 h of stroke onset. For a complete overview of the inclusion and exclusion criteria, we refer to the study protocol [21]. Patients are planned to be recruited in about 80 hospitals in 9 European countries over a period of about 4 years. To increase the generalisability of the findings, these countries are distributed across Europe and include Estonia, Germany, Greece, Hungary, Italy, the Netherlands, Norway, Poland, and the UK. For the same reason, the trial will recruit patients both in academic and regional hospitals (Table 1, Fig. 1).

**Table 1:** Baseline characteristics

	All	Paracetamol	Control	Metoclopramide	Control	Ceftriaxone	Control
Total patients randomised							
Age (years)	Mean (SD)	Mean (SD)	Mean (SD)	Mean (SD)	Mean (SD)	Mean (SD)	Mean (SD)
Sex, male (%)	*n* (%)	*n* (%)	*n* (%)	*n* (%)	*n* (%)	*n* (%)	*n* (%)
Premorbid mRS [/6]	Median [IQR]	Median [IQR]	Median [IQR]	Median [IQR]	Median [IQR]	Median [IQR]	Median [IQR]
Ethnicity, white (%)	*n* (%)	*n* (%)	*n* (%)	*n* (%)	*n* (%)	*n* (%)	*n* (%)
**Medical history (%)**							
Atrial fibrillation	*n* (%)	*n* (%)	*n* (%)	*n* (%)	*n* (%)	*n* (%)	*n* (%)
Hypercholesterolaemia	*n* (%)	*n* (%)	*n* (%)	*n* (%)	*n* (%)	*n* (%)	*n* (%)
Hypertension	*n* (%)	*n* (%)	*n* (%)	*n* (%)	*n* (%)	*n* (%)	*n* (%)
Diabetes mellitus	*n* (%)	*n* (%)	*n* (%)	*n* (%)	*n* (%)	*n* (%)	*n* (%)
Obstructive pulmonary disease	*n* (%)	*n* (%)	*n* (%)	*n* (%)	*n* (%)	*n* (%)	*n* (%)
Previous stroke	*n* (%)	*n* (%)	*n* (%)	*n* (%)	*n* (%)	*n* (%)	*n* (%)
Immunocompromised	*n* (%)	*n* (%)	*n* (%)	*n* (%)	*n* (%)	*n* (%)	*n* (%)
**Smoking, current**							
Never	*n* (%)	*n* (%)	*n* (%)	*n* (%)	*n* (%)	*n* (%)	*n* (%)
Ever	*n* (%)	*n* (%)	*n* (%)	*n* (%)	*n* (%)	*n* (%)	*n* (%)
Currently	*n* (%)	*n* (%)	*n* (%)	*n* (%)	*n* (%)	*n* (%)	*n* (%)
**Pre-stroke method of food intake**							
Normal food	*n* (%)	*n* (%)	*n* (%)	*n* (%)	*n* (%)	*n* (%)	*n* (%)
Oral softened food or fluids only	*n* (%)	*n* (%)	*n* (%)	*n* (%)	*n* (%)	*n* (%)	*n* (%)
Nasogastric tube	*n* (%)	*n* (%)	*n* (%)	*n* (%)	*n* (%)	*n* (%)	*n* (%)
Percutaneous endoscopic gastrostomy	*n* (%)	*n* (%)	*n* (%)	*n* (%)	*n* (%)	*n* (%)	*n* (%)
Intravenous only	*n* (%)	*n* (%)	*n* (%)	*n* (%)	*n* (%)	*n* (%)	*n* (%)
**Use of drugs 3 days before randomisation**							
Paracetamol	*n* (%)	*n* (%)	*n* (%)	*n* (%)	*n* (%)	*n* (%)	*n* (%)
Metoclopramide	*n* (%)	*n* (%)	*n* (%)	*n* (%)	*n* (%)	*n* (%)	*n* (%)
Ceftriaxone	*n* (%)	*n* (%)	*n* (%)	*n* (%)	*n* (%)	*n* (%)	*n* (%)
Time, onset to randomisation (min)	Mean (SD)	Mean (SD)	Mean (SD)	Mean (SD)	Mean (SD)	Mean (SD)	Mean (SD)
**Stroke type (%)**							
Ischaemic stroke	*n* (%)	*n* (%)	*n* (%)	*n* (%)	*n* (%)	*n* (%)	*n* (%)
Intracerebral haemorrhage	*n* (%)	*n* (%)	*n* (%)	*n* (%)	*n* (%)	*n* (%)	*n* (%)
Other diagnosis	*n* (%)	*n* (%)	*n* (%)	*n* (%)	*n* (%)	*n* (%)	*n* (%)
NIHSS (/42)	Mean (SD)	Mean (SD)	Mean (SD)	Mean (SD)	Mean (SD)	Mean (SD)	Mean (SD)
Systolic BP (mmHg)	Mean (SD)	Mean (SD)	Mean (SD)	Mean (SD)	Mean (SD)	Mean (SD)	Mean (SD)
Diastolic BP (mmHg)	Mean (SD)	Mean (SD)	Mean (SD)	Mean (SD)	Mean (SD)	Mean (SD)	Mean (SD)
Heart rate (bpm)	Mean (SD)	Mean (SD)	Mean (SD)	Mean (SD)	Mean (SD)	Mean (SD)	Mean (SD)
Body temperature (°C)	Mean (SD)	Mean (SD)	Mean (SD)	Mean (SD)	Mean (SD)	Mean (SD)	Mean (SD)
**Acute stroke treatment (%)**							
Intravenous thrombolysis	*n* (%)	*n* (%)	*n* (%)	*n* (%)	*n* (%)	*n* (%)	*n* (%)
Mechanical thrombectomy	*n* (%)	*n* (%)	*n* (%)	*n* (%)	*n* (%)	*n* (%)	*n* (%)

Data are *n* (%) or median [IQR]. *mRS* modified Rankin Scale, *NIHSS* National Institutes of Health Stroke Scale, *BP* blood pressure

## Statistical analysis

### Primary outcome

The primary outcome measure is the score on the modified Rankin Scale (mRS) at 90 days (± 14 days). The mRS is an ordinal scale ranging from 0 to 6 [22]. The mRS assessment at 90 days will be during a hospital/home visit or by telephone, and the assessment or a report thereof will be recorded using a digital video camera. Three blinded raters will view the videotape and adjudicate a score on the mRS.

### Primary outcome analysis

**Fig. 2:**
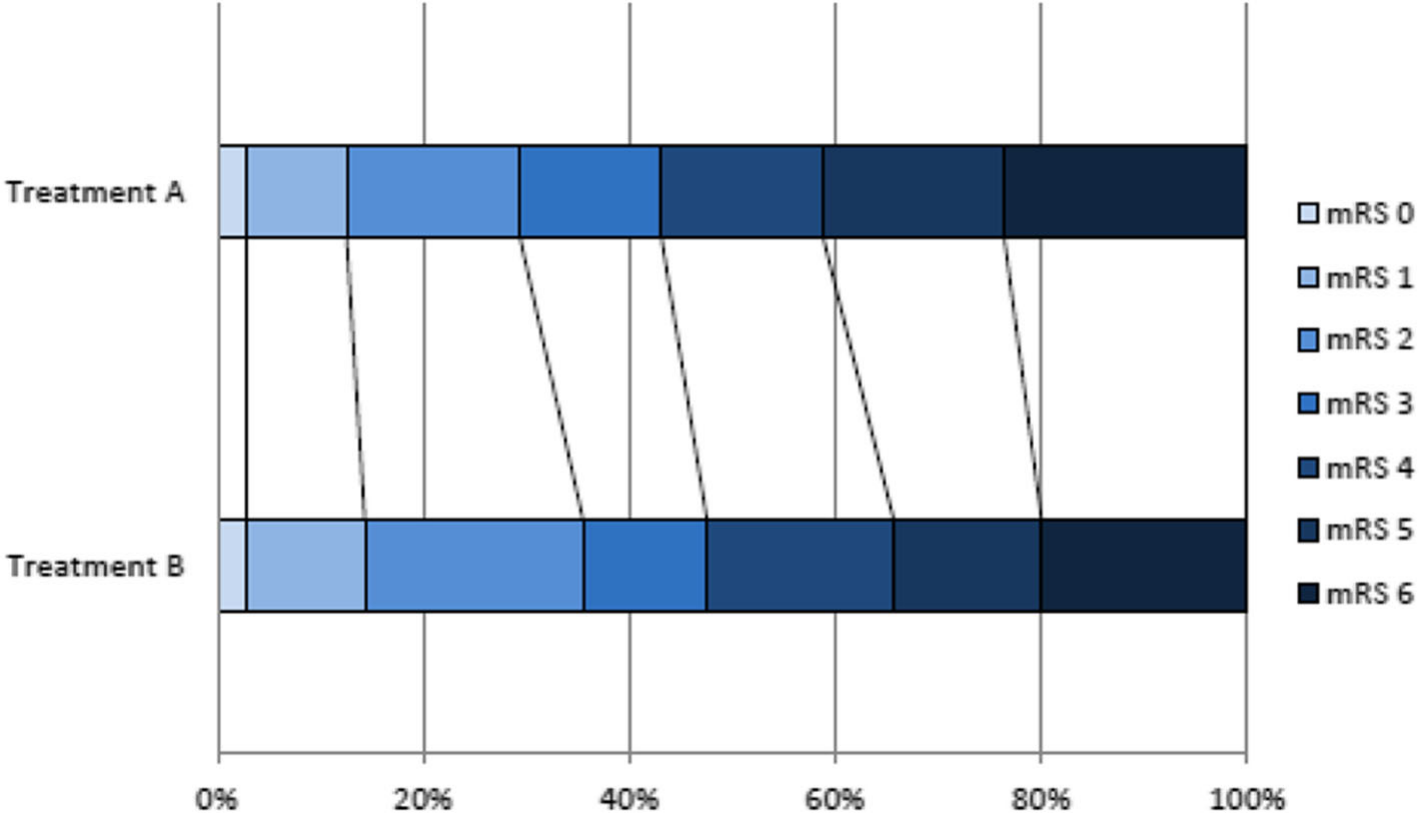
Distribution of modified Rankin Scale for each intervention using median mRS value for each participant. Example of a distribution of the modified Rankin Score at 3 months. The figure is an example, with dummy treatments and scores

For each patient, a median mRS score will be calculated from the three mRS scores obtained through centralised adjudications by raters who are blinded to treatment allocation. The use of three scores increases the precision in scoring and statistical power as compared to a single mRS assessment [23]. The primary effect estimate will be the difference in the mRS scores between the active treatment group and controls assessed using ordinal logistic regression, and will be expressed as an odds ratio with 95% confidence interval [24]. The primary analysis will be performed on all randomised patients with a valid mRS score at 90 days. The distribution of the mRS scores will be shown as a figure (Fig. 2). Three separate primary analyses will be performed for each intervention vs. their respective controls (e.g. metoclopramide vs. non-metoclopramide). The primary analyses will be adjusted for stratification (country), minimisation (age, sex, stroke type, stroke severity, diabetes), and other baseline prognostic (e.g. premorbid mRS, atrial fibrillation, reperfusion treatment [alteplase and/or thrombectomy], time from onset to randomisation) factors, and treatment allocation for the other two strata of the trial (Table 2).

**Table 2:** Primary outcome. Analyses are adjusted except where stated

	Analysis	Paracetamol	Control	DIM or OR (95% CI	Metoclopramide	Control	DIM or OR (95% CI	Ceftriaxone	Control	DIM or OR (95% CI
mRS, median	aOLR	Median [IQR]	Median [IQR]	aOR (95% CI)	Median [IQR]	Median [IQR]	aOR (95% CI)	Median [IQR]	Median [IQR]	aOR (95% CI)
**Sensitivity analyses**										
mRS, unadjusted	OLR	Median [IQR]	Median [IQR]	OR (95% CI)	Median [IQR]	Median [IQR]	OR (95% CI)	Median [IQR]	Median [IQR]	OR (95% CI)
mRS, imputed	aOLR	Median [IQR]	Median [IQR]	aOR (95% CI)	Median [IQR]	Median [IQR]	aOR (95% CI)	Median [IQR]	Median [IQR]	aOR (95% CI)
mRS, mean	aMLR	Mean (SD)	Mean (SD)	aDIM (95% CI)	Mean (SD)	Mean (SD)	aDIM (95% CI)	Mean (SD)	Mean (SD)	aDIM (95% CI)
mRS > 2	aBLR	*n* (%)	*n* (%)	aOR (95% CI)	*n* (%)	*n* (%)	aOR (95% CI)	*n* (%)	*n* (%)	aOR (95% CI)
Death	aCPHR	*n* (%)	*n* (%)	aHR (95% CI)	*n* (%)	*n* (%)	aHR (95% CI)	*n* (%)	*n* (%)	aHR (95% CI)

Data are *n* (%), median [IQR], and mean (SD). *aDIM* adjusted difference in means, *aHR* adjusted hazards ratio, *aOR* adjusted odds ratio. Comparison by adjusted ordinal logistic regression (aOLR), multiple linear regression (aMLR), cox proportional hazards regression (CPHR), or adjusted binary logistic regression (aBLR)

### Primary outcome subgroup analysis

Comparison of the effect of the three intervention groups vs. their respective controls on the primary outcome will be performed in the following pre-specified subgroups (assuming sufficient numbers in each subgroup) with assessment of interaction between treatment and the minimisation factors (these subgroup analyses are considered hypothesis-generating) (Table 3):

- Age (≤ 75, > 75 years);
- Sex (male, female);
- Stroke type (ischaemic stroke, intracerebral haemorrhage);
- Stroke severity (NIHSS 6–12, > 12);
- Diabetes mellitus (yes, no).

**Table 3:** Subgroup analysis—shown as forest plot. Adjusted analysis with interaction term

	Paracetamol	Control	aOR (95% CI)	Interaction *P*	Metoclopramide	Control	aOR (95% CI)	Interaction *P*	Ceftriaxone	Control	aOR (95% CI)	Interaction *P*
**Age**				+				+				+
Age < 75 years	*n*/*N* (%)	*n*/*N* (%)	aOR (95% CI)		*n*/*N* (%)	*n*/*N* (%)	aOR (95% CI)		*n*/*N* (%)	*n*/*N* (%)	aOR (95% CI)	
Age > 75 years	*n*/*N* (%)	*n*/*N* (%)	aOR (95% CI)		*n*/*N* (%)	*n*/*N* (%)	aOR (95% CI)		*n*/*N* (%)	*n*/*N* (%)	aOR (95% CI)	
**Sex**				+				+				+
Male	*n*/*N* (%)	*n*/*N* (%)	aOR (95% CI)		*n*/*N* (%)	*n*/*N* (%)	aOR (95% CI)		*n*/*N* (%)	*n*/*N* (%)	aOR (95% CI)	
Female	*n*/*N* (%)	*n*/*N* (%)	aOR (95% CI)		*n*/*N* (%)	*n*/*N* (%)	aOR (95% CI)		*n*/*N* (%)	*n*/*N* (%)	aOR (95% CI)	
**Stroke type**				+				+				+
Ischaemic stroke	*n*/*N* (%)	*n*/*N* (%)	aOR (95% CI)		*n*/*N* (%)	*n*/*N* (%)	aOR (95% CI)		*n*/*N* (%)	*n*/*N* (%)	aOR (95% CI)	
Intracerebral haemorrhage	*n*/*N* (%)	*n*/*N* (%)	aOR (95% CI)		*n*/*N* (%)	*n*/*N* (%)	aOR (95% CI)		*n*/*N* (%)	*n*/*N* (%)	aOR (95% CI)	
Other diagnosis	*n*/*N* (%)	*n*/*N* (%)	aOR (95% CI)		*n*/*N* (%)	*n*/*N* (%)	aOR (95% CI)		*n*/*N* (%)	*n*/*N* (%)	aOR (95% CI)	
**Stroke severity**				+				+				+
NIHSS 6–12	*n*/*N* (%)	*n*/*N* (%)	aOR (95% CI)		*n*/*N* (%)	*n*/*N* (%)	aOR (95% CI)		*n*/*N* (%)	*n*/*N* (%)	aOR (95% CI)	
NIHSS > 12	*n*/*N* (%)	*n*/*N* (%)	aOR (95% CI)		*n*/*N* (%)	*n*/*N* (%)	aOR (95% CI)		*n*/*N* (%)	*n*/*N* (%)	aOR (95% CI)	
**Diabetes mellitus**				+				+				+
Yes	*n*/*N* (%)	*n*/*N* (%)	aOR (95% CI)		*n*/*N* (%)	*n*/*N* (%)	aOR (95% CI)		*n*/*N* (%)	*n*/*N* (%)	aOR (95% CI)	
No	*n*/*N* (%)	*n*/*N* (%)	aOR (95% CI)		*n*/*N* (%)	*n*/*N* (%)	aOR (95% CI)		*n*/*N* (%)	*n*/*N* (%)	aOR (95% CI)	
**Atrial fibrillation**				+				+				+
Yes	*n*/*N* (%)	*n*/*N* (%)	aOR (95% CI)		*n*/*N* (%)	*n*/*N* (%)	aOR (95% CI)		*n*/*N* (%)	*n*/*N* (%)	aOR (95% CI)	
No	*n*/*N* (%)	*n*/*N* (%)	aOR (95% CI)		*n*/*N* (%)	*n*/*N* (%)	aOR (95% CI)		*n*/*N* (%)	*n*/*N* (%)	aOR (95% CI)	
**Pre-stroke mRS**				+				+				+
0	*n*/*N* (%)	*n*/*N* (%)	aOR (95% CI)		*n*/*N* (%)	*n*/*N* (%)	aOR (95% CI)		*n*/*N* (%)	*n*/*N* (%)	aOR (95% CI)	
> 0	*n*/*N* (%)	*n*/*N* (%)	aOR (95% CI)		*n*/*N* (%)	*n*/*N* (%)	aOR (95% CI)		*n*/*N* (%)	*n*/*N* (%)	aOR (95% CI)	
**Treatment with alteplase**				+				+				+
Yes	*n*/*N* (%)	*n*/*N* (%)	aOR (95% CI)		*n*/*N* (%)	*n*/*N* (%)	aOR (95% CI)		*n*/*N* (%)	*n*/*N* (%)	aOR (95% CI)	
No	*n*/*N* (%)	*n*/*N* (%)	aOR (95% CI)		*n*/*N* (%)	*n*/*N* (%)	aOR (95% CI)		*n*/*N* (%)	*n*/*N* (%)	aOR (95% CI)	
**Thrombectomy**				+				+				+
Yes	*n*/*N* (%)	*n*/*N* (%)	aOR (95% CI)		*n*/*N* (%)	*n*/*N* (%)	aOR (95% CI)		*n*/*N* (%)	*n*/*N* (%)	aOR (95% CI)	
No	*n*/*N* (%)	*n*/*N* (%)	aOR (95% CI)		*n*/*N* (%)	*n*/*N* (%)	aOR (95% CI)		*n*/*N* (%)	*n*/*N* (%)	aOR (95% CI)	
**Time to treatment**				+				+				+
< 6 h	*n*/*N* (%)	*n*/*N* (%)	aOR (95% CI)		*n*/*N* (%)	*n*/*N* (%)	aOR (95% CI)		*n*/*N* (%)	*n*/*N* (%)	aOR (95% CI)	
6–12 h	*n*/*N* (%)	*n*/*N* (%)	aOR (95% CI)		*n*/*N* (%)	*n*/*N* (%)	aOR (95% CI)		*n*/*N* (%)	*n*/*N* (%)	aOR (95% CI)	
12–24 h	*n*/*N* (%)	*n*/*N* (%)	aOR (95% CI)		*n*/*N* (%)	*n*/*N* (%)	aOR (95% CI)		*n*/*N* (%)	*n*/*N* (%)	aOR (95% CI)	
**Treatment allocation to other treatment strata**				+				+				+
Paracetamol	–	–	–	–	*n*/*N* (%)	*n*/*N* (%)	aOR (95% CI)		*n*/*N* (%)	*n*/*N* (%)	aOR (95% CI)	
Metoclopramide	*n*/*N* (%)	*n*/*N* (%)	aOR (95% CI)		–	–	–	–	*n*/*N* (%)	*n*/*N* (%)	aOR (95% CI)	
Ceftriaxone	*n*/*N* (%)	*n*/*N* (%)	aOR (95% CI)		*n*/*N* (%)	*n*/*N* (%)	aOR (95% CI)		–	–	–	–

Data are *n*/*N* (%). *aOR* adjusted odds ratio. Comparison by adjusted ordinal logistic regression with adjustment for an interaction term. This table will be presented as forest plots in the final publication

In addition, the interaction between treatment and other baseline factors will be assessed:

- Presence of atrial fibrillation (yes, no);
- Pre-stroke mRS score (0, > 0);
- Reperfusion treatment (alteplase and/or mechanical thrombectomy);
- Time to treatment (< 6, ≥ 6 h < 12 h, ≥ 12 h);
- Treatment allocation for the other two trial strata (paracetamol—active, control; ceftriaxone—active, control; metoclopramide—active, control). Since the study is not powered to detect interactions between the three interventions, these interactions will be investigated in secondary analyses.

### Sensitivity analyses

Four sensitivity analyses of the mRS will also be performed: unadjusted ordinal logistic regression, adjusted analysis of mRS following regression imputation of missing data, multiple linear regression on the mean mRS score for each participant, and binary logistic regression on mRS > 2.

### Secondary outcomes

The following secondary outcomes will be assessed at 7 days (± 1 day) or at discharge, if earlier:

- Infections in the first 7 days (± 1 day; frequency, type, and *Clostridium difficile* infections). Infections will be categorised as diagnosed by the clinician and as judged by an independent adjudication committee (masked to treatment allocation);
- Third generation cephalosporin resistance in the first 7 days (± 1 day), detected as part of routine clinical practice;
- Antimicrobial use during the first 7 days, converted to units of defined daily doses according to the classification of the WHO Anatomical Therapeutic Chemical Classification System with Defined Daily Doses Index;
- Serious adverse events (SAEs) in the first 7 days;
- In a subgroup of patients: presence of Extended-Spectrum Beta-Lactamase (ESBL)-producing bacteria as detected by PCR in a rectal swab at day 7 (± 1 day, or at discharge, if earlier).

The following secondary outcomes will be assessed at 90 days (± 14 days) (Table 4):

- Death;
- Unfavourable functional outcome, defined as mRS 3 to 6;
- Disability assessed with the score on the Barthel Index (BI);
- Cognition assessed with the Montreal Cognitive Assessment (MoCA);
- Quality of life assessed with the EuroQol 5D-5L (EQ-5D-5L) and EQ-visual analogue scale (EQ-VAS);
- Home time: the number of nights among the first 90 since stroke onset that are spent in the patient’s own home or a relative’s home. Resource use will be censored at 90 days. Where final follow-up occurs earlier, the last known placement will be extrapolated to 90 days;
- Patient location over first 90 days (± 14 days): hospital, rehabilitation service, chronic nursing facility, and home.

**Table 4:** Secondary outcome assessment at 90 days

	Analysis	Paracetamol	Control	OR (95% CI)	Metoclopramide	Control	OR (95% CI)	Ceftriaxone	Control	OR (95% CI)
**mRS, median**										
Ischaemic stroke	aOLR	Median [IQR]	Median [IQR]	aOR (95% CI)	Median [IQR]	Median [IQR]	aOR (95% CI)	Median [IQR]	Median [IQR]	aOR (95% CI)
Intracerebral haemorrhage	aOLR	Median [IQR]	Median [IQR]	aOR (95% CI)	Median [IQR]	Median [IQR]	aOR (95% CI)	Median [IQR]	Median [IQR]	aOR (95% CI)
Other diagnosis	aOLR	Median [IQR]	Median [IQR]	aOR (95% CI)	Median [IQR]	Median [IQR]	aOR (95% CI)	Median [IQR]	Median [IQR]	aOR (95% CI)
**Mortality**	aCPHR	*n* (%)	*n* (%)	aHR (95% CI)	*n* (%)	*n* (%)	aHR (95% CI)	*n* (%)	*n* (%)	aHR (95% CI)
**mRS, unfavourable outcome**	aBLR	*n* (%)	*n* (%)	aOR (95% CI)	*n* (%)	*n* (%)	aOR (95% CI)	*n* (%)	*n* (%)	aOR (95% CI)
**Patient location**	aOLR			aOR (95% CI)			aOR (95% CI)			aOR (95% CI)
Hospital		*n* (%)	*n* (%)		*n* (%)	*n* (%)		*n* (%)	*n* (%)	
Rehabilitation service		*n* (%)	*n* (%)		*n* (%)	*n* (%)		*n* (%)	*n* (%)	
Nursing home		*n* (%)	*n* (%)		*n* (%)	*n* (%)		*n* (%)	*n* (%)	
Home		*n* (%)	*n* (%)		*n* (%)	*n* (%)		*n* (%)	*n* (%)	
Home time (no. of days)	aMLR	Mean (SD)	Mean (SD)	aDIM (95% CI)	Mean (SD)	Mean (SD)	aDIM (95% CI)	Mean (SD)	Mean (SD)	aDIM (95% CI)
**Questionnaires**										
Barthel Index	aMLR	Mean (SD)	Mean (SD)	aDIM (95% CI)	Mean (SD)	Mean (SD)	aDIM (95% CI)	Mean (SD)	Mean (SD)	aDIM (95% CI)
MoCA	aMLR	Mean (SD)	Mean (SD)	aDIM (95% CI)	Mean (SD)	Mean (SD)	aDIM (95% CI)	Mean (SD)	Mean (SD)	aDIM (95% CI)
EQ-5D-5L	aMLR	Mean (SD)	Mean (SD)	aDIM (95% CI)	Mean (SD)	Mean (SD)	aDIM (95% CI)	Mean (SD)	Mean (SD)	aDIM (95% CI)
EQ-VAS	aMLR	Mean (SD)	Mean (SD)	aDIM (95% CI)	Mean (SD)	Mean (SD)	aDIM (95% CI)	Mean (SD)	Mean (SD)	aDIM (95% CI)

Data are *n* (%) or median [IQR]. *aDIM* adjusted difference in means, *aOR* adjusted odds ratio, *aHR* adjusted hazards ratio, *mRS* modified Rankin Scale, *MoCA* Montreal Cognitive Assessment, *EQ-5D-5L* EuroQol 5D-5L, *EQ-VAS* EuroQol-visual analogue scale. Comparison by adjusted ordinal logistic regression (aOLR), cox proportional hazards regression (aCPHR), or multiple linear regression (aMLR)

### Analysis of secondary outcomes

Binary logistic regression will be used for binary outcomes (e.g. mRS > 2). Cox proportional hazards regression will be used for time to events (e.g. death). Ordinal logistic regression will be used for ordered categorical data (e.g. mRS). Multiple linear regression will be used for continuous outcomes (e.g. BI, EQ-VAS). Patients with missing outcome data will be excluded from the analysis.

### Missing data and death

Patients without a primary outcome assessment at 90 ± 14 days will be considered as a lost to follow-up. The total amount of patients who are lost to follow-up will be recorded and calculated for each treatment arm. The primary analysis will be performed on all randomised patients with a valid mRS score at 90 days. In a sensitivity analysis, missing mRS data will be imputed using multiple regression-based imputation.

For the secondary outcome measures (Barthel Index, MoCA, EQ-5D-5L, EQ-VAS), patients who die will be assigned a value one unit worse than any living value. This way, patients who die cannot be given a score similar to the worst score of patients who are alive, and it ensures that all patients will be included in the analysis. Potential scores, with worst with dead added, are as follows:

- Modified Rankin Scale (mRS), 0 to 5 with death = 6;
- Barthel Index (BI), 100 to 0 with death = − 5;
- EuroQol 5D-5L (EQ-5D-5L), − 0.5 to 1 with death = 0;
- EuroQol visual analogue scale (EQ-VAS), 0 to 100 with death = − 1;
- Montreal cognitive assessment (MoCA), 0 to 30 with death = − 1.

### Safety outcomes

In the first 7 days after randomisation, all SAEs will be reported and described by duration (start and stop dates), severity, outcome, treatment, and relation to the investigational medical product (IMP), or if unrelated, the cause. All SAEs will be tabulated per treatment stratum. In addition, any SAE occurring between day 7 and the end of follow-up on day 90 (± 14 days) for which a causal relationship between the IMP and the SAE is considered at least a reasonable possibility (i.e. SARs and SUSARs) should be reported as other SAEs.

### Treatment restrictions

The presence of any treatment restriction will be recorded at baseline and during the hospital phase, and classified as (1) do not resuscitate, (2) do not intubate and ventilate, (3) withhold other treatments that may prolong life, (4) withhold food, (5) withhold fluids, and (6) palliation (e.g. with morphine or a benzodiazepine). Any combination of these strategies is possible. The primary study will report on the frequency of each treatment restriction, and further analyses on this topic will be published in future subgroup analyses.

### Minimising bias

PRECIOUS is an open-label clinical trial, and both patients and treating physicians are therefore aware of the assigned treatment. Knowledge of treatment allocation can influence outcome assessment, and unblinded trials like PRECIOUS are therefore at risk of detection bias. In addition, despite its apparent simplicity, assessment of the score on the mRS has been associated with considerable inter-observer variability, especially in multi-centre studies, and may therefore affect trial power and treatment effect size. In PRECIOUS, these two major issues are minimised through (1) online training and certification of outcome assessors via a link on the PRECIOUS website and (2) central outcome assessment by three blinded adjudicators based on digital video recordings of the 90-day outcome interviews. This central adjudication by trained adjudicators offers several benefits [23]:

1. Blinding is assured;
2. Standardisation is possible across multiple regions and cultures;
3. Statistical power is enhanced through the use of three repeated assessments;
4. The estimate of treatment effect size is restored (since statistical noise leads to underestimation);
5. It provides independent validation of the information that is collected, thereby minimising the risk of fraud;
6. Site staff perform to a higher standard when aware that there will be review or audit of their activity.

In addition, the risk of bias is reduced by performing the statistical analyses according to the intention-to-treat principle and adjusting for the minimisation factors, other relevant baseline characteristics, and treatment allocation for the other two strata of the trial.

## Statistical principles

### Confidence intervals and *P* values

Analyses will be two-sided *P* < 0.05 with 95% confidence intervals presented. The trial is testing the effect of the interventions on mRS, and analyses in subgroups and on other outcomes are considered hypothesis-generating. Hence, no adjustment will be made for multiplicity of testing.

### Alpha spending

The Data Monitoring Committee performs safety assessments using the Haybittle-Peto boundary rule (*P* < 0.001); hence, no significant spending of alpha will occur during the trial. All analyses will be two-tailed, and *P* values of < 0.05 will denote statistical significance; 95% confidence intervals will be provided. Adjustment for multiple comparisons will not be performed, but all contrasts will be declared.

### Compliance

Compliance with allocated treatment will be tabulated. For each of the three study drugs, the number of received dosages will be calculated (maximum of four for ceftriaxone, twelve for metoclopramide, and sixteen for paracetamol). The number of patients who received the first dosage within the time window of 24 h will also be presented; if the dosage was not given within 24 h, the reason will be given (withdrawn informed consent, death, human error, other reason).

### Analysis populations

All efficacy analyses will be performed on the intention-to-treat population. The robustness of the primary and key secondary analyses will be assessed in the per-protocol population. Safety analyses will be performed on the safety population.

The following population definitions will be used:

▪ Intention-to-treat in primary efficacy analysis: all randomised participants who received any study medication and with a valid mRS score recorded at 90 days.

▪ Intention-to-treat in primary safety analysis: all randomised participants with a vital status recorded at 90 days.

▪ Per-protocol: all participants in the intention-to-treat population who are deemed to have no major protocol violations that could interfere with the objectives of the study.

Patients with protocol violations in trial eligibility will be included in the intention-to-treat population, but excluded in the per-protocol analysis. Patients who withdrew informed consent before initiating treatment will be excluded from analysis. If (per accident) multiple randomisations are performed for a single patient, the result of the first randomisation will be used.

## Current status

The trial received approval from the central Medical Ethics Committee of the University Medical Center Utrecht, the Netherlands, on 3 February 2016. The Dutch National Competent Authority (Centrale Commissie Mensgebonden Onderzoek (CCMO)) declared to have no objection against the execution of the clinical trial within the Netherlands on 17 November 2015. In addition, the national (and local, if applicable) medical ethical committees and competent authorities of the other 8 participating countries have approved the trial. The first patient was included in May 2016. The analysis and reporting of the trial will be in accordance with CONSORT guidelines. After publication of the trial, to promote the independent re-use of PRECIOUS data, a coded dataset will be made available in a public data repository within 18 months of the final follow-up of the last patient. Coded data will also be included in the Virtual International Stroke Trials Archive (VISTA).

## Supplementary information

**Additional file 1: Table S1.** Protocol violations in eligibility. Data are n (%). mRS, modified Rankin Scale.

**Additional file 2: Table S2.** Compliance and cross-over in first 7 days. Data are n (%). Comparisons made by binary logistic regression.

**Additional file 3: Table S3.** Secondary outcomes and treatment restrictions at 7 days. mRS, modified Rankin Scale. Data are n (%) or median [IQR]. aOR: adjusted odds ratio. Comparison by adjusted ordinal logistic regression (aOLR) or binary logistic regression (aBLR). * Converted to units of defined daily doses according to the classification of the WHO Anatomical Therapeutic Chemical Classification System with Defined Daily Doses (DDD) Index.

**Additional file 4: Table S4.** Overview of safety. Data are n (%). SAE, Severe Adverse Event; SAR, Severe Adverse Reaction; SUSAR, Severe Unexpected Serious Adverse Reaction. Comparisons made by binary logistic regression.

**Additional file 5.** List of PRECIOUS partners.

## Data Availability

The details of the study protocol have been published earlier [20]. After publication of the trial, to promote the independent re-use of PRECIOUS data, a coded dataset will be made available in a public data repository within 18 months of the final follow-up of the last patient. Coded data will also be included in VISTA.

## Abbreviations

BI: Barthel Index
BLR: Binary logistic regression
BP: Blood pressure
CCMO: Centrale Commissie Mensgebonden Onderzoek
CONSORT: Consolidated Standards of Reporting Trials
CPHR: Cox proportional hazards regression
DIM: Difference in means
ESBL: Extended-Spectrum Beta-Lactamase
EQ-5D-5L: EuroQol 5D-5L
EQ-VAS: EQ-visual analogue scale
GCP: Good Clinical Practice
DDD: Daily defined dose
DSMB: Data and Safety Monitoring Board
HR: Hazard ratio
ICH: International Conference on Harmonisation
IMP: Investigational medicinal product
IQR: Interquartile range
MD: Medical doctor
MLR: Multiple linear regression
MoCA: Montreal Cognitive Assessment
mRS: Modified Rankin Scale
NIHSS: National Institutes of Health Stroke Scale
NSTU: Nottingham Stroke Trial Unit
OLR: Ordinal logistic regression
OR: Odds ratio
PhD: Doctor of philosophy
PCR: Polymerase chain reaction
PRECIOUS: PREvention of Complications to Improve OUtcome in elderly patients with acute Stroke
PROBE: Prospective randomised open blinded end-point
SAE: Serious adverse event
SAP: Statistical analysis plan
SAR: Serious adverse reaction
SD: Standard deviation
SUSAR: Suspected unexpected serious adverse reaction
UMC: University Medical Center
UNOTT: University of Nottingham
VISTA: Virtual International Stroke Trials Archive
WHO: World Health Organisation
