# Supplementary material for: PRECIOUS: PREvention of Complications to Improve OUtcome in elderly patients with acute Stroke—statistical analysis plan of a randomised, open, phase III, clinical trial with blinded outcome assessment
Source: Trials. 2020 Oct 26;21:884. doi: 10.1186/s13063-020-04717-0 (PMC7586648; doi:10.1186/s13063-020-04717-0)
Supplement: Supplementary file 1 — Additional file 1: Table S1. Protocol violations in eligibility. Data are n (%). mRS, modified Rankin Scale. [file 13063_2020_4717_MOESM1_ESM.docx]

Supplement Table 1. Protocol violations in eligibility

|  | Paracetamol | Control | Metoclopramide | Control | Ceftriaxone | Control |
| --- | --- | --- | --- | --- | --- | --- |
|  | N | N | N | N | N | N |
| Other diagnosis than stroke | n (%) | n (%) | n (%) | n (%) | n (%) | n (%) |
| NIHSS score of ≤5 | n (%) | n (%) | n (%) | n (%) | n (%) | n (%) |
| Age ≤65 years | n (%) | n (%) | n (%) | n (%) | n (%) | n (%) |
| Start treatment >24 hours | n (%) | n (%) | n (%) | n (%) | n (%) | n (%) |
| Inclusion with active infection requiring antibiotic treatment | n (%) | n (%) | n (%) | n (%) | n (%) | n (%) |
| Pre-stroke mRS ≥4 | n (%) | n (%) | n (%) | n (%) | n (%) | n (%) |
| Death is imminent | n (%) | n (%) | n (%) | n (%) | n (%) | n (%) |
| Inclusion in treatment arm despite contra-indication | n (%) | n (%) | n (%) | n (%) | n (%) | n (%) |

Data are n (%). mRS, modified Rankin Scale.
