# Supplementary material for: PRECIOUS: PREvention of Complications to Improve OUtcome in elderly patients with acute Stroke—statistical analysis plan of a randomised, open, phase III, clinical trial with blinded outcome assessment
Source: Trials. 2020 Oct 26;21:884. doi: 10.1186/s13063-020-04717-0 (PMC7586648; doi:10.1186/s13063-020-04717-0)
Supplement: Supplementary file 2 — Additional file 2: Table S2. Compliance and cross-over in first 7 days. Data are n (%). Comparisons made by binary logistic regression. [file 13063_2020_4717_MOESM2_ESM.docx]

**Supplement Table 2. Compliance and cross-over in first 7 days**

|  | Paracetamol | Control | P | Metoclopramide | Control | P | Ceftriaxone | Control | P |
| --- | --- | --- | --- | --- | --- | --- | --- | --- | --- |
|  | N | N |  | N | N |  | N | N |  |
| Received all allocated dosages | n (%) | - | - | n (%) | - | - | n (%) | - | - |
| Received 75-99% of dosages | n (%) | - | - | n (%) | - | - | n (%) | - | - |
| Received 50-<75% of dosages | n (%) | - | - | n (%) | - | - | n (%) | - | - |
| Received 25-<50% of dosages | n (%) | - | - | n (%) | - | - | n (%) | - | - |
| Received 0-<25% of dosages | n (%) | - | - | n (%) | - | - | n (%) | - | - |
| Received any antibiotic drug | n (%) | n (%) |  | n (%) | n (%) |  | n (%) | n (%) |  |
| Received any antipyretic drug | n (%) | n (%) |  | n (%) | n (%) |  | n (%) | n (%) |  |
| Received any antipyretic drug for four days at least once | n (%) | n (%) |  | n (%) | n (%) |  | n (%) | n (%) |  |
| Received any anti-emetic drug | n (%) | n (%) |  | n (%) | n (%) |  | n (%) | n (%) |  |
| Received any anti-emetic drug for four days at least once | n (%) | n (%) |  | n (%) | n (%) |  | n (%) | n (%) |  |
