# Supplementary material for: PRECIOUS: PREvention of Complications to Improve OUtcome in elderly patients with acute Stroke—statistical analysis plan of a randomised, open, phase III, clinical trial with blinded outcome assessment
Source: Trials. 2020 Oct 26;21:884. doi: 10.1186/s13063-020-04717-0 (PMC7586648; doi:10.1186/s13063-020-04717-0)
Supplement: Supplementary file 3 — Additional file 3: Table S3. Secondary outcomes and treatment restrictions at 7 days. mRS, modified Rankin Scale. Data are n (%) or median [IQR]. aOR: adjusted odds ratio. Comparison by adjusted ordinal logistic regression (aOLR) or binary logistic regression (aBLR). * Converted to units of defined daily doses according to the classification of the WHO Anatomical Therapeutic Chemical Classification System with Defined Daily Doses (DDD) Index. [file 13063_2020_4717_MOESM3_ESM.docx]

**Supplement Table 3. Secondary outcomes and treatment restrictions at 7 days**

|  | Analysis | Paracetamol | Control | OR (95% CI) | Metoclopramide | Control | OR (95% CI) | Ceftriaxone | Control | OR (95% CI) |
| --- | --- | --- | --- | --- | --- | --- | --- | --- | --- | --- |
| **mRS, median** |  |  |  |  |  |  |  |  |  |  |
| All patients | aOLR | Median [IQR] | Median [IQR] | aOR (95% CI) | Median [IQR] | Median [IQR] | aOR (95% CI) | Median [IQR] | Median [IQR] | aOR (95% CI) |
| Ischemic stroke | aOLR | Median [IQR] | Median [IQR] | aOR (95% CI) | Median [IQR] | Median [IQR] | aOR (95% CI) | Median [IQR] | Median [IQR] | aOR (95% CI) |
| Haemorrhagic stroke | aOLR | Median [IQR] | Median [IQR] | aOR (95% CI) | Median [IQR] | Median [IQR] | aOR (95% CI) | Median [IQR] | Median [IQR] | aOR (95% CI) |
| Mortality at 7 days | aBLR | n (%) | n (%) | aOR (95% CI) | n (%) | n (%) | aOR (95% CI) | n (%) | n (%) | aOR (95% CI) |
| Any treatment restriction | - | n (%) | n (%) | - | n (%) | n (%) | - | n (%) | n (%) | - |
| **Infection** |  |  |  |  |  |  |  |  |  |  |
| All infections | aBLR | n (%) | n (%) | aOR (95% CI) | n (%) | n (%) | aOR (95% CI) | n (%) | n (%) | aOR (95% CI) |
| Pneumonia | aBLR | n (%) | n (%) | aOR (95% CI) | n (%) | n (%) | aOR (95% CI) | n (%) | n (%) | aOR (95% CI) |
| Urinary tract infection | aBLR | n (%) | n (%) | aOR (95% CI) | n (%) | n (%) | aOR (95% CI) | n (%) | n (%) | aOR (95% CI) |
| Other infections | aBLR | n (%) | n (%) | aOR (95% CI) | n (%) | n (%) | aOR (95% CI) | n (%) | n (%) | aOR (95% CI) |
| **Infections based on expert panel** |  |  |  |  |  |  |  |  |  |  |
| All infections | aBLR | n (%) | n (%) | aOR (95% CI) | n (%) | n (%) | aOR (95% CI) | n (%) | n (%) | aOR (95% CI) |
| Pneumonia | aBLR | n (%) | n (%) | aOR (95% CI) | n (%) | n (%) | aOR (95% CI) | n (%) | n (%) | aOR (95% CI) |
| Urinary tract infection | aBLR | n (%) | n (%) | aOR (95% CI) | n (%) | n (%) | aOR (95% CI) | n (%) | n (%) | aOR (95% CI) |
| Other infections | aBLR | n (%) | n (%) | aOR (95% CI) | n (%) | n (%) | aOR (95% CI) | n (%) | n (%) | aOR (95% CI) |
| **Antimicrobial use and resistance** |  |  |  |  |  |  |  |  |  |  |
| 3rd generation cephalosporin resistance | aBLR | n (%) | n (%) | aOR (95% CI) | n (%) | n (%) | aOR (95% CI) | n (%) | n (%) | aOR (95% CI) |
| Antimicrobial use during first 7 days* |  | DDD | DDD | - | DDD | DDD | - | DDD | DDD | - |

mRS, modified Rankin Scale. Data are n (%) or median [IQR]. aOR: adjusted odds ratio. Comparison by adjusted ordinal logistic regression (aOLR) or binary logistic regression (aBLR).

* Converted to units of defined daily doses according to the classification of the WHO Anatomical Therapeutic Chemical Classification System with Defined Daily Doses (DDD) Index;
