# Supplementary material for: PRECIOUS: PREvention of Complications to Improve OUtcome in elderly patients with acute Stroke—statistical analysis plan of a randomised, open, phase III, clinical trial with blinded outcome assessment
Source: Trials. 2020 Oct 26;21:884. doi: 10.1186/s13063-020-04717-0 (PMC7586648; doi:10.1186/s13063-020-04717-0)
Supplement: Supplementary file 4 — Additional file 4: Table S4. Overview of safety. Data are n (%). SAE, Severe Adverse Event; SAR, Severe Adverse Reaction; SUSAR, Severe Unexpected Serious Adverse Reaction. Comparisons made by binary logistic regression. [file 13063_2020_4717_MOESM4_ESM.docx]

**Supplement Table 4. Overview of safety**

|  | Paracetamol | Control | P | Metoclopramide | Control | P | Ceftriaxone | Control | P |
| --- | --- | --- | --- | --- | --- | --- | --- | --- | --- |
| Infections diagnosed by physician | n (%) | n (%) |  | n (%) | n (%) |  | n (%) | n (%) |  |
| - Pneumonia | n (%) | n (%) |  | n (%) | n (%) |  | n (%) | n (%) |  |
| - Urinary tract infection |  |  |  |  |  |  |  |  |  |
| - Other infection | n (%) | n (%) |  | n (%) | n (%) |  | n (%) | n (%) |  |
| Pneumonia diagnosed by an independent adjudication committee | n (%) | n (%) |  | n (%) | n (%) |  | n (%) | n (%) |  |
| *Clostridium difficile* infection of the gastro-intestinal tract | n (%) | n (%) |  | n (%) | n (%) |  | n (%) | n (%) |  |
| Infection with a ceftriaxone resistant micro-organism | n (%) | n (%) |  | n (%) | n (%) |  | n (%) | n (%) |  |
| Liver function disturbance or liver failure | n (%) | n (%) |  | n (%) | n (%) |  | n (%) | n (%) |  |
| Allergic or hypersensitivity reaction  **Other SAEs**: | n (%) | n (%) |  | n (%) | n (%) |  | n (%) | n (%) |  |
| Total amount of SAEs | n (%) | n (%) |  | n (%) | n (%) |  | n (%) | n (%) |  |
| Total amount of related SAEs (SARs or SUSARs) | n (%) | n (%) |  | n (%) | n (%) |  | n (%) | n (%) |  |
| Total amount of SUSARs | n (%) | n (%) |  | n (%) | n (%) |  | n (%) | n (%) |  |

Data are n (%). SAE, Severe Adverse Event; SAR, Severe Adverse Reaction; SUSAR, Severe Unexpected Serious Adverse Reaction. Comparisons made by binary logistic regression**.**
