# Supplementary material for: PRECIOUS: PREvention of Complications to Improve OUtcome in elderly patients with acute Stroke—statistical analysis plan of a randomised, open, phase III, clinical trial with blinded outcome assessment
Source: Trials. 2020 Oct 26;21:884. doi: 10.1186/s13063-020-04717-0 (PMC7586648; doi:10.1186/s13063-020-04717-0)
Supplement: Supplementary file 5 — Additional file 5. List of PRECIOUS partners. [file 13063_2020_4717_MOESM5_ESM.docx]

List of PRECIOUS partners

| **Affiliation** | **Investigator(s)** |
| --- | --- |
| Department of Neurology and Neurosurgery, Brain Center, University Medical Center Utrecht, Utrecht University, Utrecht, The Netherlands. | Hendrik Reinink, Jeroen C de Jonge, H Bart van der Worp |
| Stroke Trials Unit, Division of Clinical Neuroscience, University of Nottingham, Nottingham, United Kingdom | Philip M Bath |
| Department of Neurology, Academic Medical Center, Amsterdam Neuroscience, Amsterdam, The Netherlands | Diederik van de Beek |
| Department of Internal Medicine, Oslo, University Hospital, Oslo, Norway | Eivind Berge |
| Department of Neurology and Stroke Unit, ASST di Mantova, Mantua, Italy. | Alfonso Ciccone |
| Department of Neurology, University of Debrecen, Debrecen, Hungary | Laszlo Csiba |
| European Clinical Research Infrastructure Network (ECRIN), Paris, France | Jacques Demotes |
| Department of Neurology, Erasmus MC, University Medical Center, Rotterdam, The Netherlands | Diederik W Dippel |
| Department of Neurology and Neurosurgery, University of Tartu, Tartu, Estonia | Janika Korv |
| 2^nd^ Department of Neurology, Institute of Psychiatry and Neurology, Warsaw , Poland | Iwona Kurkowska-Jastrzebska |
| Institute of Cardiovascular and Medical sciences, University of Glasgow, Glasgow, United Kingdom | Kennedy R Lees |
| Division of Clinical Neurosciences, Centre for Clinical Brain Sciences, University of Edinburgh, Edinburgh, United Kingdom | Malcolm R Macleod |
| Department of Medicine, Larissa University Hospital, University of Thessaly, Larissa, Greece | George Ntaios |
| Stroke Alliance for Europe (SAFE), Brussels, Belgium | Gary Randall |
| Department of Neurology, Center for Clinical Neurosciences, University Medical Center Hamburg-Eppendorf, Hamburg, Germany. | Götz Thomalla |
